# Genome-Wide Association Study and Gene-Based Analysis of Participants With Hemophilia A and Inhibitors in the My Life, Our Future Research Repository

**DOI:** 10.3389/fmed.2022.903838

**Published:** 2022-06-23

**Authors:** Samuel Lessard, Chunla He, Deepak K. Rajpal, Katherine Klinger, Christine Loh, Tim Harris, Jennifer Dumont

**Affiliations:** ^1^Sanofi S.A., Framingham, MA, United States; ^2^American Thrombosis and Hemostasis Network, Rochester, NY, United States; ^3^Bioverativ, a Sanofi Company, Waltham, MA, United States; ^4^Sanofi S.A., Cambridge, MA, United States

**Keywords:** genome-wide association study, inhibitors, humans, whole-genome sequencing, major histocompatibility complex, hemophilia A

## Abstract

**Introduction:**

Up to 30% of individuals with hemophilia A develop inhibitors to replacement factor VIII (FVIII), rendering the treatment ineffective. The underlying mechanism of inhibitor development remains poorly understood. The My Life, Our Future Research Repository (MLOF RR) has gathered *F8* and *F9* mutational information, phenotypic data, and biological material from over 11,000 participants with hemophilia A (HA) and B as well as carriers enrolled across US hemophilia treatment centers, including over 5,000 whole-genome sequences. Identifying genes associated with inhibitors may contribute to our understanding of why certain patients develop those neutralizing antibodies.

**Aim and Methods:**

Here, we performed a genome-wide association study and gene-based analyses to identify genes associated with inhibitors in participants with HA from the MLOF RR.

**Results:**

We identify a genome-wide significant association within the human leukocyte antigen (HLA) locus in participants with HA with *F8* intronic inversions. HLA typing revealed independent associations with the HLA alleles major histocompatibility complex, class II, DR beta 1 (HLA DRB1*15:01) and major histocompatibility complex, class II, DQ beta 1 (DQB1*03:03). Variant aggregation tests further identified low-frequency variants within *GRID2IP* (glutamate receptor, ionotropic, delta 2 [*GRID2*] interacting protein 1) significantly associated with inhibitors.

**Conclusion:**

Overall, our study confirms the association of DRB1*15:01 with FVIII inhibitors and identifies a novel association of DQB1*03:03 in individuals with HA carrying intronic inversions of *F8*. In addition, our results implicate *GRID2IP*, encoding *GRID2*-interacting protein, with the development of inhibitors, and suggest an unrecognized role of this gene in autoimmunity.

## Introduction

Inhibitor development against factor VIII (FVIII) is the most serious complication of replacement factor therapy. Thirty percent of individuals with severe hemophilia A (HA) receiving factor replacement therapy develop inhibitors, rendering the treatment ineffective (1). The underlying basis for why some individuals develop inhibitors while others do not remains poorly understood, but risk factors include ethnic background, family history, and *F8* variant, suggesting that genetics is an important contributor (2–6). Individuals with HA with intronic inversions, large structural variants, or nonsense variants are 7–10 times more likely to develop inhibitors compared with those with milder mutations (4). Individuals with missense variants have the lowest incidence of inhibitors (<10%) presumably because they synthesize FVIII polypeptides that can induce tolerance (5). The intron 22 inversion accounts for up to 50% of mutations among individuals with severe HA and results in a truncated *F8* with inverted intron 1 through 22 (4, 7). Individuals with inhibitors are classified as low or high responders if their inhibitor titers are below or higher than 5 Bethesda units per mL (BU/mL), respectively. Individuals with high-titer inhibitors are less likely to respond to immune tolerance induction (ITI) (8). Therefore, the genetic risk factors contributing to low or high inhibitors may differ and may be dependent on the underlying pathogenic *F8* variant.

Genetic studies have been performed to investigate mechanisms and biomarkers of inhibitor development in individuals with HA, and have implicated variants in genes including *MAPK9*, *CD86*, *HMOX1*, *FCGR2A*, *IL2*, *IL10*, *TNF*, *LTA*, and *CTLA4* (9–22). The largest study included over 13,000 single-nucleotide polymorphisms from 1,081 genes in 833 subjects (22). In addition, human leukocyte antigen (HLA) alleles have been associated with inhibitor risk, including major histocompatibility complex, class II, DR beta 1 (HLA DRB1*15:01) and major histocompatibility complex, class II, DQ beta 1 (HLA DQB1*06:02) (17, 19, 23–25).

Genome-wide association studies (GWAS) have become more widely used as an approach to uncover the etiology of diseases. They have some limitations, however, as they require a large sample size and may miss rare variants. In cases where multiple rare variants with small effects are likely to contribute to the disease, whole exome sequencing and gene burden analysis have been used to provide insights into clinical phenotype (26).

The My Life, Our Future Research Repository (MLOF RR) has gathered *F8* and *F9* mutational information, phenotypic data, and biological material from over 11,000 participants with hemophilia A and B as well as carriers enrolled across US hemophilia treatment centers, and whole-genome sequencing of over 5,000 genomes (27, 28).

Here, GWAS and gene-based analyses were performed in participants with HA from the MLOF RR to identify genes associated with inhibitor development.

## Materials and Methods

### My Life, Our Future Study

The MLOF program consists of a collaboration between the American Thrombosis and Hemostasis Network (ATHN), National Hemophilia Foundation, and Bloodworks Northwest, with funding from Bioverativ, a Sanofi company (27). Informed consent was obtained for inclusion of data and biological samples from a subset of participants who were chosen for whole-genome sequencing (WGS). The MLOF study included participants with hemophilia of all types and severities, and whether a causative *F8* or *F9* variant was identified or not. In this study, only the subset of participants with HA were considered. Phenotypic and demographic data, including self-reported race and ethnicity, were provided by ATHN and were collected across hemophilia treatment centers around the United States.

### Whole-Genome Sequencing

WGS of samples was conducted as part of TOPMed (Trans-Omics for Precision Medicine),^1^ and data collection, processing, and quality control are described elsewhere (27, 29). WGS data were obtained through the database of Genotypes and Phenotypes (dbGaP, phs001515.v1.p1) as a processed Variant Call Format file as well as mapped reads (compressed alignment files). Variants that did not pass quality control or those that were monomorphic were filtered out within the MLOF RR cohort, and 119,015,152 variants remained. Samples duplicated or with sex mismatch were removed (N = 10). We produced a genetic relationship matrix using the PLINK 2.0-make-king option (30) and estimated principal components (PCs) using PC-AiR (31). We generated a final genetic relationship matrix using PC-Relate and the PCs from PC-AiR to account for population structure (32).

### Inhibitor Definition

We defined participants with HA as having a history of inhibitors (cases) if they had active inhibitors and currently prescribed bypassing agents or ITI, or if they had a previous history of inhibitors and were not currently prescribed bypassing agents or ITI. We defined controls (participants with HA without inhibitors) as having no history of usage of bypassing agents or ITI and status reported as “no history of inhibitors,” or if presence of inhibitors was ruled out. Participants with high-titer inhibitors had a peak inhibitor titer of >5 BU/mL as measured by the Bethesda inhibitor assay. The low-titer inhibitors group was not defined because we could not discriminate between low-titer participants or participants with missing or non-exhaustive BU measures. Participants with unknown inhibitor status were excluded.

### Cohort Definitions

Given the difference in inhibitor prevalence between ethnic groups (3, 6), and to reduce confounding due to population structure, we separated the MLOF RR cohort by genetic ancestry. For each subgroup, we excluded participants if any of their first 2 genetic PCs were >3 standard deviations away from the group mean. The largest subgroup was individuals who reported “White” (European) ethnicity and not “Hispanic, Latino/a, or Spanish origin” (*N* = 2,246), and we performed discovery analyses on this subset of individuals. We attempted replication of significant associations in the 2 other largest subgroups from this dataset, namely “Black or African American” (*N* = 310) and “Hispanic, Latino/a, or Spanish origin” (*N* = 488), excluding participants with mild hemophilia A in the replication cohorts due to small sample size. We restricted all analyses to male participants with HA because only 1 female participant with genetic data had a history of inhibitors. Total numbers of cases and controls included are reported in Table 1.

**TABLE 1:** HA inhibitor cohort definitions and case-control counts.

Analysis	European ancestry (discovery)			Black or African American (replication)		Hispanic, Latino/a, Spanish (replication)	
			
	Controls, *N*	Cases, *N*	MAF threshold,%	Controls, *N*	Cases, *N*	Controls, *N*	Cases, *N*
All inhibitor	1699	306	1.0	198	77	237	87
High-titer inhibitor	1699	110	2.3	198	35	237	32
*F8* inversion	411	142	1.8	61	39	72	51

In single-variant association analyses, only variants with an MAF >1% and ≥5 expected minor allele counts in cases were included, i.e., MAF ≥ 5/(2*N_cases_). The column “MAF threshold” reports the approximate minimum allele frequency threshold that satisfies both conditions for each of the 3 main analyses. A similar approach was used for variant aggregation tests, where only genes were included if the aggregated expected minor allele count was ≥5. The number of cases and controls represent the number included in the association analyses after filtering (methods).

### Single-Variant Analysis

We performed single-variant association tests on inhibitor status as a binary phenotype using logistic mixed-model score tests implemented in the R (3.6.1) package generalized linear mixed-model association tests (GMMAT) (v1.2.0) (33), adjusting for age, *F8* variant, hemophilia A severity, sequencing center, and the first 3 PCs. Pathogenic *F8* variant types were defined as large inversions, large structural variants [>50 base pairs (bp)], splice-site, nonsense, frameshift, and other [small (<50 bp) in-frame, synonymous, missense, and untranslated region variants]. Disease severity was defined as mild (>5 to <50% FVIII), moderate (1 to <5% FVIII), or severe (<1% FVIII) hemophilia A. We also performed association tests restricted to the subset of participants with intronic inversions using GMMAT, adjusting for age, sequencing center, and the first 3 PCs. The genetic relationship matrix estimated from PC-related was used as input for GMMAT. Analyses were restricted to variants with a minor allele frequency (MAF) >1% and ≥5 expected variant counts in cases. Variants with a *P* value <5 × 10^–8^ were considered statistically significant. We performed association tests for three different phenotype definitions: (1) all-titer inhibitors, (2) high-titer inhibitors (BU > 5), and (3) all-titer inhibitors restricted to participants with intronic inversions. All three case definitions were compared to non-inhibitor controls, restricting to participants with HA with intronic inversion in analysis 3. We generated locus plots using LocusZoom (34).

### Replication and Meta-Analysis (Single Variant)

As the score test does not provide effect size estimates, we reanalyzed all variants With *P* < 1 × 10^−7^ in the European-ancestry discovery analysis using Wald tests implemented in GMMAT, which provides effect size estimates and standard errors. Logistic mixed models were adjusted for covariates. Then, we attempted to replicate those associations in the replication (i.e., non-European ancestry) cohorts using Wald tests, adjusting for *F8* mutation type, sequencing center, age, and the first 3 PCs. Given the smaller sample sizes of non-European ancestry cohorts, mutation type was more broadly defined as presence or absence of large *F8* structural variants or intronic inversions in those cohorts.

We used inverse variance meta-analysis as implemented in the R package *meta* to combine results for the discovery and replication analyses (35).

### Variant Aggregation Analysis

We conducted gene-based analyses using variant-set mixed-model association tests (SMMAT) (36) and restricted the analysis to variants with an MAF < 5% and annotated as missense or predicted to have a high impact on protein-coding genes as defined using SnpEff 4.1 (37). We only considered genes with ≥ 3 polymorphic variants and an aggregated expected minor allele count in cases ≥ 5. Gene-based tests were adjusted for covariates as in the single-variant analysis.

### Replication and Meta-Analysis (Variant Aggregation Analysis)

Replication tests of genes significantly associated with inhibitors in the discovery analysis were attempted in the replication cohorts using SMMAT and meta analyzed each cohort using smmat.meta included in the GMMAT package, including covariates, as described above.

### Human Leukocyte Antigen Typing

We typed HLA alleles based on the WGS data using HLA*LA (bioconda v1.0) (38) and performed 4-digit HLA allele association analysis in R by logistic regression, adjusting for covariates as in the single-variant analysis. We performed HLA allele association for all participants with inhibitors and a subgroup of participants with intronic inversion. Each HLA allele was tested separately based on the presence or absence of the allele. HLA alleles with a Bonferroni-corrected *P* value < 0.05 were significant. We attempted to replicate significant association in the non-European cohorts. For participants with an intronic inversion, we further tested each HLA allele after conditioning on the presence or absence of DRB1*15:01.

### FinnGen Phenome-Wide Association Study

We retrieved genetic associations for *GRID2IP* (glutamate receptor, ionotropic, delta 2 [*GRID2*] interacting protein 1) in the FinnGen dataset through the FinnGen PheWeb portal (http://r6.finngen.fi, release 6). We report all associations with *P* < 1 × 10^–4^.

### Expression Quantitative Trait Loci (eQTL) and Gene Expression

We retrieved expression quantitative trait loci (eQTL) for *IGLV* (immunoglobulin lambda variable) cluster genes from the Genotype-Tissue Expression portal (version 8). Gene expression of *GRID2IP* was assessed in the European Bioinformatic Institute (EBI) Expression Atlas through the Open Targets portal (39). We also assessed colocalization between GWAS and eQTL studies through the Open Targets Genetics portal.^2^

### Association With Clinical Phenotypes

We tested the association between significant variants identified in the association analyses and the following clinical phenotypes: age at inhibitor diagnosis, peak inhibitor titer, number of bleeds in the last 3 years, successful ITI, and length of ITI treatment. We tested the association between quantitative and binary phenotypes using linear and logistic regression respectively, adjusting for age, genetic ancestry, hemophilia A severity, and *F8* variant type. Quantitative phenotypes were log-transformed prior to association testing.

## Results

### Genome-Wide Association Study of Inhibitors in Participants With HA

**FIGURE 1:**
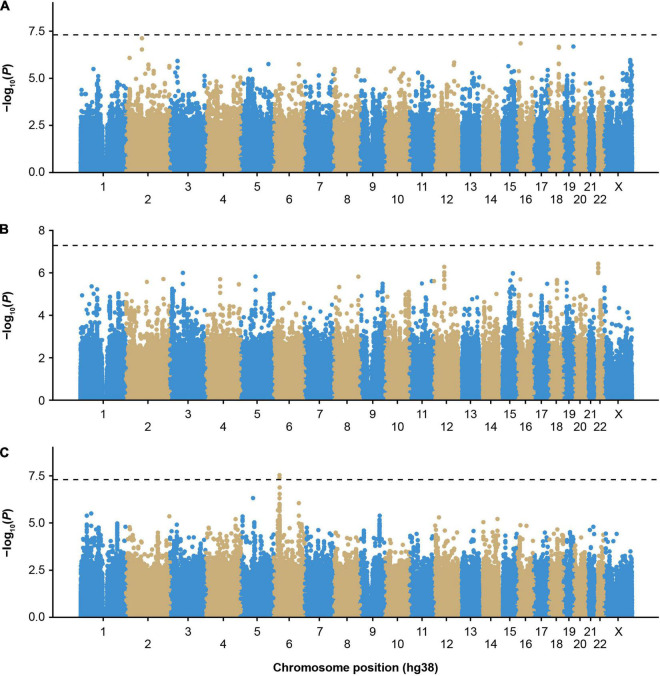
Genome-wide association study of inhibitors in European participants of the MLOF study. **(A)** Participants with all-titer inhibitor hemophilia A (*N* = 306 cases, *N* = 1,699 controls), **(B)** high-titer inhibitor participants (*N* = 110 cases, *N* = 1,699 controls), and **(C)** participants with all-titer inhibitor hemophilia A with *F8* intronic inversions (*N* = 142 cases, N = 411 controls). Colors distinguish the different chromosomes. *P*-values were calculated by performing score tests using GMMAT after fitting logistic mixed models to account for population structure and relatedness and adjusting for age, sequencing center, *F8* mutation type, severity, and the first 3 genetic principal components. *P*-values < 5 × 10^–8^ were considered significant. GMMAT, generalized linear mixed-model association tests; hg, human genome; MLOF, My Life, Our Future.

We first performed single-variant GWAS of participants with inhibitors in the MLOF RR using logistic mixed models implemented in GMMAT, focusing on participants of European ancestry, the largest subgroup (Supplementary Table 1). No variant reached genome-wide significance (Figure 1A and Supplementary Figure 1). The most significant variant was rs62158559 (*P* = 7.6 × 10^–8^), downstream of *FOXI3*. We tested the association of all variants with an association *P* value <1 × 10^–7^ with inhibitors in independent MLOF RR subgroups of Black or African American and Hispanic, Latino/a, or Spanish origin using Wald tests and performed inverse variance meta-analysis (Supplementary Table 2). No variant reached significance.

To reduce heterogeneity that may be due to differing genetic risk factors between participants with low- (≤5 BU) or high-titer inhibitors (>5 BU), we performed a GWAS including only high-titer inhibitor participants (Figure 1B and Supplementary Figure 2), and no loci reached genome-wide significance (*P* < 5 × 10^–8^). However, we detected an association trend for a cluster of variants in linkage disequilibrium (cut-off: r^2^ > 0.8) near the chromosome 22 *IGLV* cluster (Supplementary Figures 3, 4 and Supplementary Table 2). The top variant, rs5756720 (*P* = 3.6 × 10^–7^), has low frequency in European ancestry participants (MAF = 2.3%) but reached a frequency of 8% in high-titer inhibitor participants.

**FIGURE 2:**
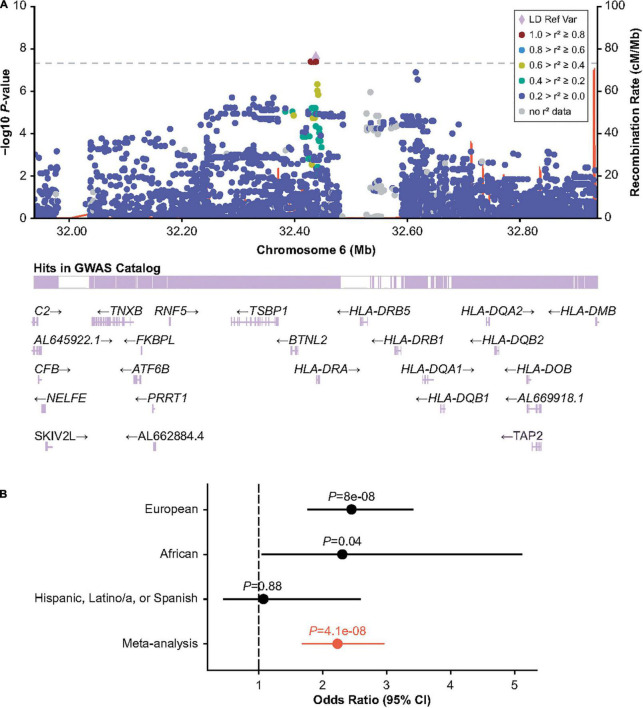
Association of human leukocyte antigen (HLA) region variant 6:32438468_CA/C with inhibitors in participants with HA with intronic *F8* inversions. **(A)** LocusZoom plot showing GMMAT score test *P-value* within the HLA region. **(B)** Wald-test *P-value* and odds ratios for the discovery (European ancestry) analysis, and replication analyses in individuals of Black or African American and Hispanic, Latino/a, or Spanish origin. Odds ratios are calculated for the CA allele. Meta-analysis odds ratio and *P* values were calculated by inverse variance meta-analysis. CI, confidence interval; GMMAT, generalized linear mixed-model association tests; GWAS, genome-wide association study; LD Ref Var, linkage disequilibrium reference variant; HA, hemophilia A.

The HLA region has previously been implicated in inhibitor formation (17–19, 23–25). We hypothesized that different HLA alleles, as well as other genetic risk factors, may be implicated in the formation of inhibitors depending on the underlying *F8* variant. Inversions were one of the most frequent mutation types observed in MLOF (27%–40% of all participants), consistent with that of common hemophilia A variants (4, 7, 40). Therefore, we performed a GWAS of inhibitors (all titers) restricting to participants with intronic inversions (Figure 1C and Supplementary Figure 5) and observed a significant association for variant Chr6:32438468_CA/C (*P* = 4.1 × 10^–8^), located near *HLA-DRA* (Figure 2A), which we replicated in the Black or African American subgroup (*P* = 0.040) (Figure 2B). Participants with intronic inversions carrying the CA allele had an increased inhibitor risk (meta-analysis odds ratio [OR] = 2.3 [1.7–3.0]) compared to those with the C allele.

### DRB1*15:01 and DQB1*03:03 Are Associated With Inhibitors in Participants With HA

To understand which HLA gene was implicated in inhibitor formation, we typed HLA alleles using HLA*LA (38). Presence of the DRB1*15:01 allele was significantly associated with increased inhibitor risk after multiple testing correction (European ancestry *P* = 1.7 × 10^–4^, meta-analysis *P* = 5.6 × 10^–5^) (Table 2; Supplementary Tables 4–6). The association with DRB1*15:01 was slightly stronger when stratifying for participants with *F8* inversions (European *P* = 2.0 × 10^–5^, meta-analysis *P* = 2.8 × 10^–5^). DQB1*06:02 was also significantly associated with increased inhibitors within this subgroup (European ancestry *P* = 7.5 × 10^–5^, meta-analysis *P* = 8.3 × 10^–5^) but was not significant after conditioning for DRB1*15:01 in participants with intronic inversions (*P* > 0.05). Interestingly, we detected a secondary independent association with DQB1*03:03 after conditioning on DRB1*15:01 (*P* = 3.1 × 10^–4^) (Table 2).

**TABLE 2:** Human leukocyte antigen (HLA) alleles associated with inhibitors.

Analysis	HLA gene	Allele	European ancestry				Black or African American				Hispanic, Latino/a, Spanish				Meta-analysis	
				
			OR (95% CI)	*P-value*	N	Freq (%)	OR (95% CI)	*P-value*	N	Freq (%)	OR (95% CI)	*P* value	N	Freq (%)	*P-value*	OR (95% CI)
All *F8* mutations	DRB1	15:01	1.63 (1.26–2.10)	0.00017	1987	13.1	1.09 (0.38–3.11)	0.88	274	3.6	2.21 (0.91–5.36)	0.080	324	4.6	5.6 × 10^–5^	1.63 (1.28–2.06)
Intronic inversions only	DRB1	15:01	2.20 (1.53–3.17)	2.0 × 10^–5^	551	14.5	1.50 (0.29–7.85)	0.63	100	4.5	1.09 (0.22–5.30)	0.92	123	2.8	2.8 × 10^–5^	2.10 (1.48–2.96)
Intronic inversions only	DQB1	06:02	2.11 (1.46–3.05)	7.5 × 10^–5^	551	14.2	1.38 (0.63–3.04)	0.42	100	16.5	1.43 (0.39–5.27)	0.59	123	3.3	8.3 × 10^–5^	1.92 (1.39–2.65)
Intronic inversions only, conditional on DRB1*15:01	DQB1	03:03	3.11 (1.68–5.75)	0.00031	551	4.4	1.36 (0.33–5.66)	0.68	100	5.0	1.14 (0.25–5.15)	0.86	123	3.3	9 × 10^–4^	2.45 (1.44–4.16)

We typed HLA alleles from WGS using HLA*LA. We tested each allele at 4-digit resolution separately (presence or absence) for association with inhibitors using a logistic model. We tested all alleles with a frequency >1% in European ancestry participants (N = 116 HLA alleles). We used Bonferroni correction to adjust for multiple testing (i.e., P < 4.3 × 10^–4^ for 117 tests). We attempted to replicate significant associations in the Black or African American and Hispanic, Latino/a, or Spanish cohorts. We combined P values by fixed-effect inverse variance meta-analysis. CI, confidence interval; DRB1, major histocompatibility complex, class II, DR beta 1; DQB1, major histocompatibility complex, class II, DQ beta 1; Freq, frequency; OR, odds ratio; WGS, whole-genome sequencing.

### Low-Frequency Variants in *GRID2IP* Are Associated With High-Titer Inhibitors

We performed variant aggregation tests using the efficient hybrid burden and Sequence Kernel Association Test (SKAT) implemented in SMMAT (36) to assess whether aggregating low-frequency and rare variants (MAF < 0.05) could reveal additional associations. We first tested whether we could capture the effect of *F8* mutations on inhibitor status by gene burden tests by omitting *F8* mutation type or hemophilia A severity in the regression model. *F8* mutations were associated with inhibitors (burden *P* = 1.6 × 10^–6^, efficient test *P* = 1.0 × 10^–5^) (Supplementary Table 6); small *F8* mutations were protective against inhibitors, reflecting that the participants carrying those variants are less likely to develop inhibitors compared with participants with large structural variants (Supplementary Table 7).

**FIGURE 3:**
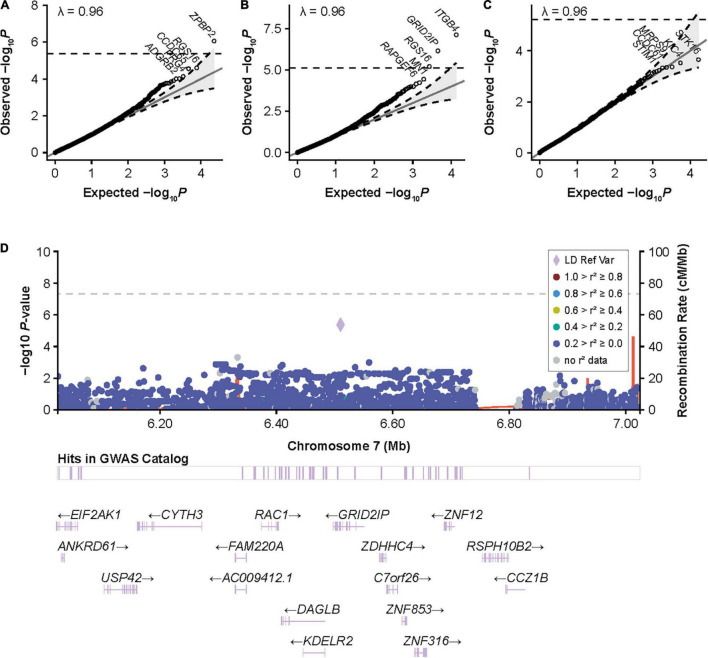
Gene-based associations with inhibitors and high-titer inhibitors in the European discovery cohort and around *GRID2IP* in participants with HA. **(A)** Quantile-quantile (QQ) plots for inhibitor status, **(B)** high-titer inhibitor status, and **(C)** inhibitor status in patients with intronic *F8* inversions in the European discovery cohort for gene-based variant aggregation tests. Association *P* values were calculated using logistic mixed-model score tests using variant-set mixed-model association tests (SMMAT), adjusting for age, sequencing center, *F8* mutation type, hemophilia A severity, and the first 3 principal components. **(D)** LocusZoom plot reporting single-variant generalized linear mixed-model association tests (GMMAT) score test *P* values of high-titer inhibitors in participants with hemophilia A around *GRID2IP*. Logistic mixed models were adjusted for age, sequencing center, *F8* mutation type, hemophilia A severity, and the first three principal components. In contrast to the main single-variant analysis with high-titer inhibitor, all variants with an allele frequency >1% were included to show the association with the top coding *GRID2IP* variant included in variant aggregation tests (rs61732374, *P* = 4 × 10^–6^). *GRID2IP*, glutamate receptor, ionotropic, delta 2 [*GRID2*] interacting protein 1; GWAS, genome-wide association study; LD Ref Var, linkage disequilibrium reference variant; HA, hemophilia A.

We next assessed whether other genes were associated with inhibitors after controlling for *F8* mutation type and severity. We identified a significant association for *ZPBP2* with all-titer inhibitors, and *ITGB4*, *GRID2IP*, and *RGS16* with high-titer inhibitors (Table 3; Figure 3; and Supplementary Tables 3, 4, 8–10). Of these 4 genes, the association with *GRID2IP* was replicated in Black or African American participants (*P* = 0.0078, meta-analysis *P* = 1.6 × 10^–8^). The strongest single-variant association within *GRID2IP* was observed for rs61732374 (Chr7:6510953_G_A, Supplementary Table 11). The rs61732374-A allele was associated with increased inhibitor risk (MAF = 2%, single-variant, Wald *P* = 2.1 × 10^–5^, OR [95% confidence interval (CI)] = 5.2 [2.4–11.0]). The same variant was nominally associated with inhibitors in Black or African American participants (MAF = 1%, Wald *P* = 0.0092, OR [95% CI] = 32.2 [2.4–439.7]; meta-analysis *P* = 1.5 × 10^–6^) but was monomorphic in individuals of Hispanic, Latino/a, or Spanish origin. rs61732374 encodes a missense variant changing arginine 504 to a cysteine.

**TABLE 3:** Significant genes associated with inhibitors by variant-aggregation tests.

Gene	European ancestry		Black or African American		Hispanic, Latino/a, Spanish		Meta-analysis	
				
	Variants, N	*P*-value	Variants, N	*P*-value	Variants, N	*P*-value	Variants, N	*P*-value
**All inhibitor (N tests: 11,509; significance threshold: 4 × 10** ^–^ **^6^)**								
*ZPBP2*	8	8.8 × 10^–7^	7	0.90	8	0.97	19	0.0017
**High-titer inhibitors (N tests: 6,737; significance threshold: 7 × 10** ^–^ **^6^)**								
*ITGB4*	77	7.6 × 10^–8^	30	0.42	16	0.44	106	4.3 × 10^–5^
*GRID2IP*	41	7.0 × 10^–7^	29	0.0078	15	0.22	70	1.6 × 10^–8^
*RGS16*	9	6.2 × 10^–6^	5	1.00	2	0.76	12	0.0037

*P-values for the SMMAT “efficient” test are reported. Only genes with ≥5 expected minor alleles in cases and ≥3 variants in the gene are reported (N = 11,509 and N = 6,737 for all and high-titer inhibitor analyses, respectively). We defined significant genes as those passing Bonferroni-corrected p-values. GRID2IP, glutamate receptor, ionotropic, delta 1 (GRID2) interacting protein 1; ITGB4, integrin subunit beta 4; RGS16, regulatory of G protein signaling 16; SMMAT, variant-set mixed-model association tests; ZPBP2, zona pellucida binding protein 2.*

### Association With Clinical Phenotypes

**FIGURE 4:**
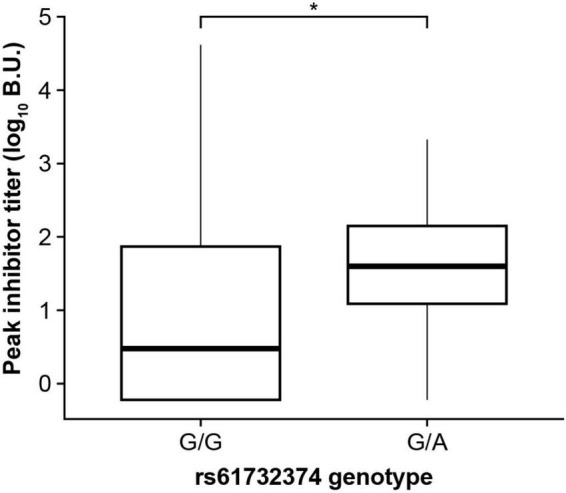
The *GRID2IP* rs61732374-A allele is associated with increased peak inhibitor titer. Significance was evaluated using a linear regression model adjusting for age, hemophilia A severity, *F8* mutation type, and genetic ancestry group. Inhibitor titers were log transformed prior to association testing. The analysis included black or African American as well as European ancestry participants with history of inhibitors (current or past) and available peak inhibitor titer information (*N* = 310). The analysis could not be performed in the Hispanic, Latino/a, or Spanish group as the A-allele was not observed in this population. BU, Bethesda Units. * *P* < 0.05.

We investigated whether DRB1*15:01, DQB1*03:03, and the *GRID2IP* rs61732374 missense variant were associated with additional clinical phenotypes in MLOF participants, including number of bleeds, peak inhibitor titers, and age at inhibitor diagnosis. We did not observe a significant association between the 2 HLA alleles and number of bleeds, inhibitor titers, or age at inhibitor diagnosis (*P* > 0.05). However, inhibitor-positive participants carrying the rs61732374-A allele displayed increased inhibitor titers (*P* = 0.017, Figure 4). In addition, 162 participants underwent ITI. We tested whether these genotypes could influence ITI success or length of treatment but did not identify significant associations (*P* > 0.05).

## Discussion

We performed a large-scale GWAS of FVIII inhibitors in the MLOF RR cohort in participants who underwent WGS. Despite comprehensive genotyping, no variant reached genome-wide significance when including all participants with HA, which suggests that our study may be underpowered to detect associations due to the relatively low number of inhibitor cases and/or disease heterogeneity. The latter is supported by the detection of significant association of a variant within the HLA region with inhibitors in participants carrying intronic inversions.

To understand which HLA alleles may contribute to inhibitor risk, we typed and performed an association analysis of HLA alleles at 4-digit resolution. We identified an association between the presence of DRB1*15:01 and increased risk of inhibitors. This association was stronger in participants with intronic inversions, in whom we identified a secondary independent association with DQB1*03:03. DRB1*15:01 has been previously identified as associated with inhibitors, particularly in participants with intron 22 inversions (23). To our knowledge, we report for the first time an association with DQB1*03:03, which was only detectable after conditioning on DRB1*15:01. In addition to increased sample size, this may partly explain why association with this allele was not detected in previous studies (23, 24, 41, 42). Nonetheless, this result will require replication in independent studies.

HLA genes identified in this study encode major histocompatibility complex class II molecules, which may contribute to inhibitor formation through presentation of *F8* peptides to CD4 T-helper cells by antigen-presenting cells. In addition, our results suggest that HLA alleles may modulate inhibitor risk (5) differently depending on the underlying *F8* mutation. Different *F8* missense variants, such as p.Arg612Cys and p.Arg2169His, are more likely to lead to inhibitor development in part because they lead to higher levels of novel binding between treatment FVIII peptides and HLA-DRB1 (43). Similar mechanisms may be at play for FVIII inversions, although individuals with HA with the intron-22 inversion may be partly tolerized to FVIII, as the full FVIII sequence remains expressed intracellularly as 2 non-secreted polypeptides (44).

The HLA variant DPB1*02:02 has been previously identified as a risk factor for inhibitor development in the MLOF study (45). The variant was not included in our analysis as its frequency was under our inclusion threshold (<1%). Outside the HLA region, we also detected a potential association with variants within the chromosome 22 *IGLV* cluster. The *IGLV* genes encode the variable region of immunoglobulins, which defines the antigen-binding site. Therefore, it is plausible that variants that modulate *IGLV* genes outside their coding sequence may influence the risk of developing neutralizing autoantibodies, although we only detected colocalizing eQTLs for *IGLV* pseudogenes (Supplementary Figure 4).

Finally, variant aggregation tests identified an association of rare variants in *ZPBP2* with inhibitors, and *ITGB4*, *GRID2IP*, and *RGS16* with high-titer inhibitors. Both *ZPBP2* and *GRID2IP* are part of loci that have been previously associated with autoimmune disease, including asthma and inflammatory bowel diseases (IBDs) (46–52). This suggests that inhibitor risk may be modulated by common loci associated with inhibitors. It is possible that the associations detected in this study are capturing the effect of common nearby variants. However, the strongest association for inhibitors around *GRID2IP* was with the low-frequency coding variant rs61732374, in weak linkage disequilibrium with nearby variants (r^2^ < 0.2) (Supplementary Figure 3). In addition, all variants included in *ZPBP2* were rare (MAF < 1%).

We observed some inflation at the tails of gene-based test quantile-quantile plots, particularly for the high-titer inhibitor analysis, although this was not reflected in the genomic control (λ = 0.96). This may be due to the imbalance between the number of case and control participants (6% high-titer inhibitor cases). Nevertheless, we were able to replicate the *GRID2IP* association in Black or African American participants. The association between inhibitors and *GRID2IP* variants was only observed in the high-titer inhibitor group. Consistently, we observed a significant association between the low-frequency *GRID2IP* missense variant, rs61732374 (Arg504Cys), and increased inhibitor titers at peak in participants with a history of inhibitors. Although our results suggest that variants in *GRID2IP* may increase the risk of developing high levels of inhibitors, we did not identify a significant impact on ITI outcomes in the subset of participants who underwent this treatment. *GRID2IP* encodes the glutamate receptor ionotropic delta 2 (*GRID2*) interacting protein, which is predominantly expressed in the brain and functions as a linker between *GRID2* and actin at the parallel fiber-Purkinje cell synapse (53). *GRID2IP* expression is strongly upregulated in peripheral blood mononuclear cells of patients with vitiligo (54). Interestingly, another *GRID2IP* missense variant, rs184043502, was strongly associated with IBDs in the FinnGen biobank (*P* = 2 × 10^–10^) (Supplementary Table 12). In addition, significant association at the *GRID2IP* locus for irritable bowel disease and white blood cell counts strongly colocalized with *GRID2IP* blood eQTLs (posterior colocalization probability > 0.97) (39, 49, 55). Together, this suggests that *GRID2IP* may have an unrecognized role in autoimmunity.

## Conclusion

In conclusion, GWAS and HLA typing revealed a significant association of variants in the HLA region, in particular HLA DRB1*15:01 and DQB1*03:03, with inhibitors in participants with *F8* intronic inversions. Our results suggest that the underlying cause of FVIII inhibitors is likely heterogeneous and dependent on the underlying *F8* pathogenic variant. Finally, we identified and replicated an association between low-frequency coding variants in *GRID2IP* and FVIII inhibitors, highlighting a potential role of the gene in anti-drug antibody development.

## Data Availability Statement

The original contributions presented in this study are included in the article/supplementary material, further inquiries can be directed to the corresponding author.

## Ethics Statement

The Coordinating Ethics Committee of the Hospital District of Helsinki and Uusimaa (HUS) approved the FinnGen study protocol Nr HUS/990/2017. MLOF phenotypic data were provided by ATHN under the WIRB Protocol #20122010 on a de-identified basis. The patients/participants provided their written informed consent to participate in this study.

## Author Contributions

SL designed the study, carried out analyses, interpreted data, performed statistical analyses, and wrote the manuscript. CH analyzed data and performed statistical analyses. KK, DR, and JD designed the study, collected and interpreted data, and wrote the manuscript. JD, TH, and CL designed the original research plan. All authors provided critical revision of the manuscript and had final approval of the manuscript for publication.

## Author Disclaimer

The content of this manuscript is solely the responsibility of the authors and does not necessarily represent the official views of the MLOF program or its partners.

## Conflict of Interest

SL, DR, KK, and JD were employed by Sanofi, DR, KK, and JD hold shares and/or stock options in the company. CL was employed by Nimbus Therapeutics. TH was employed by Repertoire Immune Medicines. The remaining author declares that the research was conducted in the absence of any commercial or financial relationships that could be construed as a potential conflict of interest. This study was funded by Sanofi (Cambridge, MA, United States). The funder had the following involvement with the study: Sanofi (and Sobi) reviewed the manuscript and Sanofi funded editorial support, provided by Ashleigh Pulkoski-Gross, Ph.D., CMPP, and Sheila Longo, Ph.D., of JK Associates Inc., part of Fishawack Health. All authors declare no other competing interests. CL and TH are former employees of Sanofi.

## Publisher’s Note

All claims expressed in this article are solely those of the authors and do not necessarily represent those of their affiliated organizations, or those of the publisher, the editors and the reviewers. Any product that may be evaluated in this article, or claim that may be made by its manufacturer, is not guaranteed or endorsed by the publisher.

## Footnotes

1
www.nhlbiwgs.org

2
http://genetics.opentargets.org/
