# Supplementary material for: Genome-Wide Association Study and Gene-Based Analysis of Participants With Hemophilia A and Inhibitors in the My Life, Our Future Research Repository
Source: Front Med (Lausanne). 2022 Jun 23;9:903838. doi: 10.3389/fmed.2022.903838 (PMC9260508; doi:10.3389/fmed.2022.903838)
Supplement: Supplementary file 2 [file Table_1.DOCX]

**SUPPLEMENTARY INFORMATION**

**Supplementary table 1.** **Frequency of *F8* mutation categories in the My Life, Our Future Research Repository (MLOF RR) cohort.**

| **Mutation type** | **European ancestry** | | **Black or African American** | | **Hispanic, Latino/a, Spanish** | | |
| --- | --- | --- | --- | --- | --- | --- | --- |
|  | **N** | **%** | N | **%** | | **N** | **%** |
| **Missense** | 1102 | 47.0 | 101 | 29.1 | | 99 | 25.8 |
| **Inversion** | 635 | 27.1 | 128 | 36.9 | | 149 | 38.9 |
| **Frameshift** | 245 | 10.5 | 37 | 10.7 | | 48 | 12.5 |
| **Nonsense** | 157 | 6.7 | 38 | 11.0 | | 27 | 7.0 |
| **Large structural changes (>50 bp)** | 73 | 3.1 | 30 | 8.6 | | 16 | 4.2 |
| **Splice-site** | 64 | 2.7 | 9 | 2.6 | | 12 | 3.1 |
| **Unknown** | 34 | 1.5 | 1 | 0.3 | | 8 | 2.1 |
| **Small in-frame structural changes (<50 bp)** | 19 | 0.8 | 2 | 0.6 | | 2 | 0.5 |
| **Synonymous** | 10 | 0.4 | 0 | 0.0 | | 22 | 5.7 |
| **Untranslated region** | 4 | 0.2 | 1 | 0.3 | | 0 | 0.0 |

bp, base pairs.

**Supplementary table 2.** **Replication of single-variant associations with *P*<1×10^-7^ in the European discovery analysis in individuals of Black or African American and Hispanic, Latino/a, or Spanish origin.** Only variants with a minor allele frequency >1% are included.

Provided in separate Excel file.

**Supplementary table 3.** **Human leukocyte antigen (HLA) allele association results for all patients with inhibitors in the European discovery cohort.** Alleles with a minor allele frequency >1% are reported. Association *P* values were calculated by logistic regression, adjusting for age, sequencing center, *F8* mutation type, hemophilia A severity, and the first 3 principal components.

Provided in separate Excel file.

**Supplementary table 4.** **Human leukocyte antigen (HLA) allele association results for patients with inhibitors with intronic inversions in the European discovery cohort.** Alleles with a minor allele frequency >1% are reported. Association *P* values were calculated by logistic regression, adjusting for age, sequencing center, and the first 3 principal components.

Provided in separate Excel file.

**Supplementary table 5.** **Human leukocyte antigen (HLA) allele association results for inhibitor patients with intronic inversions in the European discovery cohort, after conditioning for HLA DRB1*15:01 alleles.** Alleles with a minor allele frequency >1% are reported. Association *P* values were calculated by logistic regression, adjusting for age, sequencing center, and the first 3 principal components.

Provided in separate Excel file.

**Supplementary table 6. *F8* variant aggregation test results using variant-set mixed-model association tests (SMMAT) in European-ancestry (EA) participants without adjusting for *F8* mutations or severity.** Models are adjusted for age, sequencing center, and the first 3 principal components.

| **Model** | **Variants, N** | **Burden score** | **Burden *P*** | **SMMAT *P*** |
| --- | --- | --- | --- | --- |
| Full (EA) | 508 | -1832.9 | 1.6×10^-6^ | 1.0×10^-5^ |
| High titer (EA) | 476 | -1129.7 | 2.7×10^-6^ | 3.7×10^-5^ |

**Supplementary table 7.** **Association of *F8* mutations categories with inhibitor status using logistic regression.** Missense variants are used as reference.

| **Mutation type** | **European ancestry** | | | **Black or African American** | | **Hispanic, Latino/a, Spanish** | | |
| --- | --- | --- | --- | --- | --- | --- | --- | --- |
|  | **OR (95% CI)** | ***P* value** | | **OR (95% CI)** | ***P* value** | **OR (95% CI)** | | ***P* value** |
| **Frameshift** | 3.15 (2.0–4.94) | 5.30E-07 | 4.76 (1.49–15.25) | | 8.60E-03 | 1.9 (0.65–5.53) | 2.40E-01 | |
| **Inversion** | 5.39 (3.87–7.5) | 1.90E-23 | 7.99 (3.17–20.13) | | 1.00E-05 | 6.14 (2.82–13.36) | 4.80E-06 | |
| **Large structural changes (>50 bp)** | 9.99 (5.65–17.67) | 2.50E-15 | 6.25 (1.9–20.51) | | 2.50E-03 | 11.14 (3.34–37.18) | 8.80E-05 | |
| **Nonsense** | 6.63 (4.2–10.49) | 5.70E-16 | 6.88 (2.26–20.87) | | 6.70E-04 | 2.71 (0.8–9.16) | 1.10E-01 | |
| **Small in-frame structural changes (<50 bp)** | 0.92 (0.12–7.02) | 9.30E-01 | 0 (0–Inf) | | 9.90E-01 | 0 (0–Inf) | 9.80E-01 | |
| **Splice-site** | 1.91 (0.78–4.65) | 1.50E-01 | 12.5 (2.06–75.88) | | 6.10E-03 | 4.33 (0.92–20.38) | 6.30E-02 | |
| **Synonymous** | 0 (0–Inf) | 9.90E-01 | N/A | | N/A | 0.43 (0.05–3.62) | 4.40E-01 | |
| **Unknown** | 0 (0–Inf) | 9.80E-01 | 71972660.76 (0–Inf) | | 9.90E-01 | 4.33 (0.69–27.08) | 1.20E-01 | |
| **Untranslated region** | 0 (0–Inf) | 9.90E-01 | 71972660.74 (0–Inf) | | 9.90E-01 | N/A | N/A | |

bp, base pairs; CI, confidence interval; Inf, infinity; OR, odds ratio.

**Supplementary table 8.** **Variant-set mixed-model association tests (SMMAT) gene-based test results for inhibitor status in the European discovery analysis.** Only genes with at least 3 polymorphic variants and an expected minor allele count >5 in cases were included. Age, sequencing center, *F8* mutation type, hemophilia A severity, and the first 3 principal components were included as covariates. Only variants with a minor allele frequency <5% and annotated as missense or predicted to have a “high impact” on the protein sequence as defined by SnpEff were included.

Provided in separate Excel file.

**Supplementary table 9.** **Variant-set mixed-model association tests (SMMAT) gene-based test results for high-titer inhibitors in the European discovery analysis.** Only genes with at least 3 polymorphic variants and an expected minor allele count >5 in cases were included. Age, sequencing center, *F8* mutation type, hemophilia A severity, and the first 3 principal components were included as covariates. Only variants with a minor allele frequency <5% and annotated as missense or predicted to have a “high impact” on the protein sequence as defined by SnpEff were included.

Provided in separate Excel file.

**Supplementary table 10.** **Variant-set mixed-model association tests (SMMAT) gene-based test results for inhibitor status in participants with intronic inversions in the European discovery analysis.** Only genes with at least 3 polymorphic variants and an expected minor allele count >5 in cases were included. Age, sequencing center, and the first 3 principal components were included as covariates. Only variants with a minor allele frequency <5% and annotated as missense or predicted to have a “high impact” on the protein sequence as defined by SnpEff were included.

Provided in separate Excel file.

**Supplementary table** **11.** **Single variants score test results for genes significant in variant aggregation analyses.**

Provided in separate Excel file.

**Supplementary table 12.** ***GRID2IP* missense and loss-of-function variants with association *P* values <1×10^-4^ in FinnGen release 6.** Results were obtained through the FinnGen PheWeb browser.

| **Variant** | **rs ID** | **Imputation (INFO)** | **Consequence** | **Phenotype** | **N cases** | **N controls** | **MAF cases** | **MAF controls** | **OR** | ***P* value** |
| --- | --- | --- | --- | --- | --- | --- | --- | --- | --- | --- |
| 7:6503078:C:T | rs201998040 | 0.980006 | missense_variant | Other papulosquamous disorders | 157 | 252323 | 0.0191 | 0.000965 | 2.18E+08 | 2.61E-07 |
| 7:6503078:C:T | rs201998040 | 0.980006 | missense_variant | Carcinoma in situ of skin, other sites/unspecified | 125 | 260280 | 0.016 | 0.000963 | 1.69E+08 | 2.70E-05 |
| 7:6503078:C:T | rs201998040 | 0.980006 | missense_variant | Carcinoma in situ of skin, other sites/unspecified (controls excluding all cancers) | 125 | 209098 | 0.016 | 0.000955 | 3.28E+08 | 2.87E-05 |
| 7:6508277:G:T | rs184043502 | 0.984452 | missense_variant | IBD patients in KELA register | 5047 | 249705 | 0.0734 | 0.0573 | 1.345627 | 1.50E-10 |
| 7:6508277:G:T | rs184043502 | 0.984452 | missense_variant | Interstitial lung disease endpoints | 27365 | 233040 | 0.0633 | 0.0569 | 1.134747 | 1.66E-09 |
| 7:6508277:G:T | rs184043502 | 0.984452 | missense_variant | Inflammatory bowel disease, strict (require KELA) | 4611 | 249705 | 0.0724 | 0.0573 | 1.321807 | 5.99E-09 |
| 7:6508277:G:T | rs184043502 | 0.984452 | missense_variant | IBD | 7206 | 253199 | 0.0688 | 0.0573 | 1.233801 | 4.38E-08 |
| 7:6508277:G:T | rs184043502 | 0.984452 | missense_variant | UC patients in KELA register (KELA 208 prior to 1994, or ICD K51) | 3752 | 256653 | 0.072 | 0.0574 | 1.307779 | 3.14E-07 |
| 7:6508277:G:T | rs184043502 | 0.984452 | missense_variant | Crohn’s disease of large intestine | 1004 | 249705 | 0.0845 | 0.0573 | 1.674995 | 3.68E-07 |
| 7:6508277:G:T | rs184043502 | 0.984452 | missense_variant | Noninfective enteritis and colitis | 10700 | 249705 | 0.0653 | 0.0573 | 1.162694 | 1.75E-06 |
| 7:6508277:G:T | rs184043502 | 0.984452 | missense_variant | Ulcerative rectosigmoiditis | 1494 | 249705 | 0.0776 | 0.0573 | 1.451852 | 4.93E-06 |
| 7:6508277:G:T | rs184043502 | 0.984452 | missense_variant | Crohn’s disease | 2532 | 249705 | 0.0726 | 0.0573 | 1.329735 | 6.46E-06 |
| 7:6508277:G:T | rs184043502 | 0.984452 | missense_variant | Other Crohn’s disease | 1226 | 249705 | 0.0793 | 0.0573 | 1.490617 | 9.62E-06 |
| 7:6508277:G:T | rs184043502 | 0.984452 | missense_variant | Crohn’s disease NAS | 1237 | 249705 | 0.0778 | 0.0573 | 1.480115 | 1.12E-05 |
| 7:6508277:G:T | rs184043502 | 0.984452 | missense_variant | Ankylosing spondylitis | 1846 | 194289 | 0.0748 | 0.0572 | 1.366373 | 1.36E-05 |
| 7:6508277:G:T | rs184043502 | 0.984452 | missense_variant | Psoriasis | 5621 | 252323 | 0.0662 | 0.0574 | 1.19953 | 3.15E-05 |
| 7:6508277:G:T | rs184043502 | 0.984452 | missense_variant | UC (strict definition, require KELA) | 3276 | 256653 | 0.0699 | 0.0574 | 1.259632 | 3.36E-05 |
| 7:6508277:G:T | rs184043502 | 0.984452 | missense_variant | UC | 5349 | 249705 | 0.0673 | 0.0573 | 1.199194 | 3.39E-05 |
| 7:6508277:G:T | rs184043502 | 0.984452 | missense_variant | Crohn’s disease patients in KELA register (KELA code 209, or 208 with ICD-10 K50) | 1177 | 259228 | 0.0784 | 0.0575 | 1.460502 | 3.47E-05 |
| 7:6508277:G:T | rs184043502 | 0.984452 | missense_variant | Crohn’s disease (strict definition, require KELA, min 2 HDR) | 1020 | 259228 | 0.0793 | 0.0575 | 1.4821 | 6.08E-05 |
| 7:6508277:G:T | rs184043502 | 0.984452 | missense_variant | Crohn’s disease of small intestine | 1198 | 249705 | 0.0768 | 0.0573 | 1.434304 | 7.48E-05 |
| 7:6508277:G:T | rs184043502 | 0.984452 | missense_variant | Crohn’s disease (strict definition, require KELA) | 1047 | 259228 | 0.0787 | 0.0575 | 1.460049 | 8.90E-05 |
| 7:6508277:G:T | rs184043502 | 0.984452 | missense_variant | Papulosquamous disorders | 8082 | 252323 | 0.0643 | 0.0574 | 1.153222 | 9.22E-05 |
| 7:6508277:G:T | rs184043502 | 0.984452 | missense_variant | Ulcerative colitis (strict definition, require KELA, min 2 HDR) | 3135 | 256653 | 0.0694 | 0.0574 | 1.248234 | 9.57E-05 |
| 7:6510953:G:A | rs61732374 | 0.96059 | missense_variant | Wide developmental disorders | 471 | 259934 | 0.0301 | 0.0136 | 4.190123 | 1.76E-05 |

*GRID2IP*, glutamate receptor, ionotropic, delta 2 [*GRID2*] interacting protein 1; HDR, health data record; IBD, irritable bowel disease; ICD, International Classification of Diseases; MAF, minor allele frequency; NAS, non aliter specificatus; OR, odds ratio; UC, ulcerative colitis.


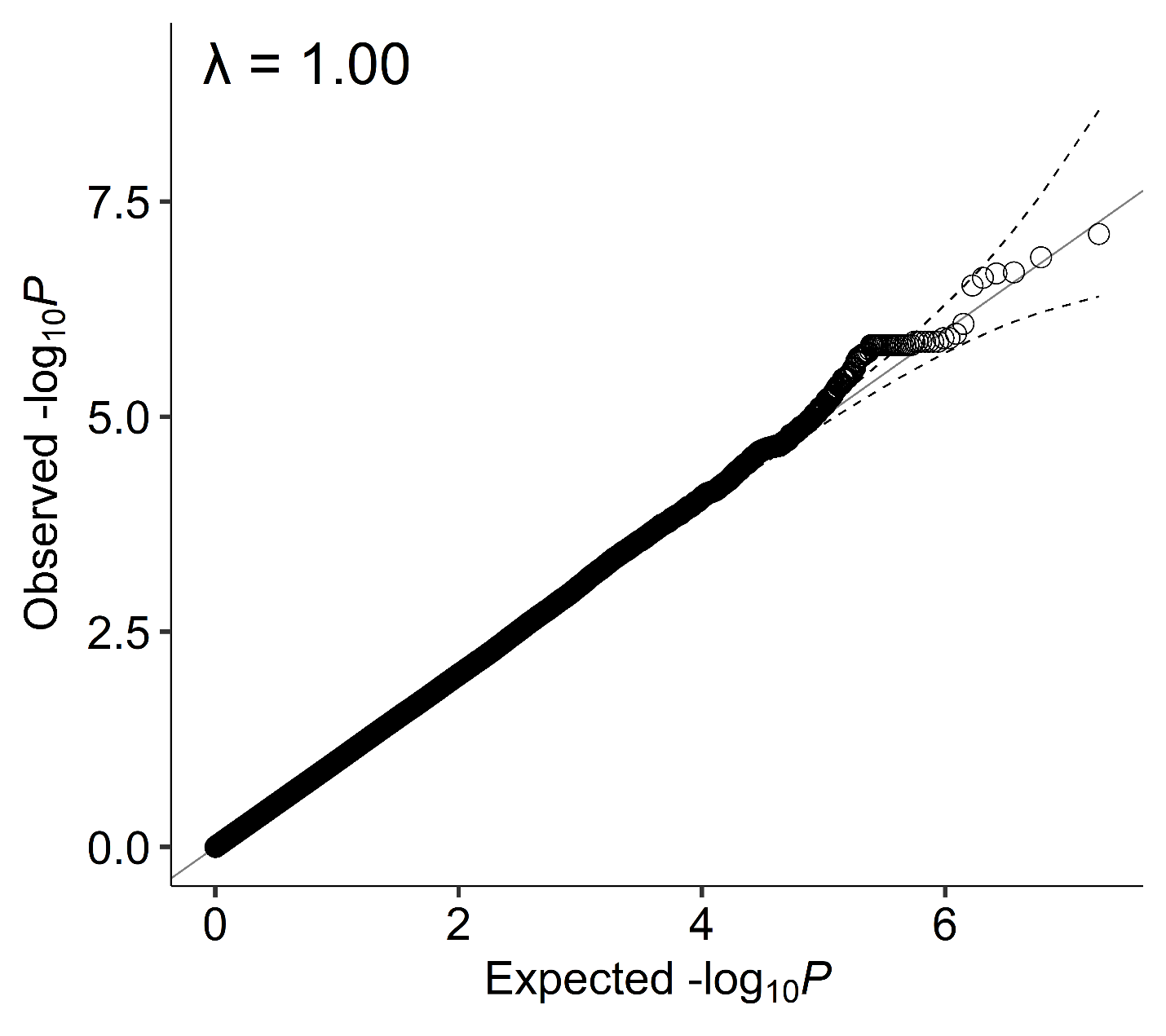


**Figure S1. Quantile-quantile (QQ) plot for inhibitor status in the European discovery cohort.** Association *P* values were calculated using logistic mixed-model score tests as implemented in generalized linear mixed-model association tests (GMMAT), adjusting for age, sequencing center, *F8* mutation type, hemophilia A severity, and the first 3 principal components.


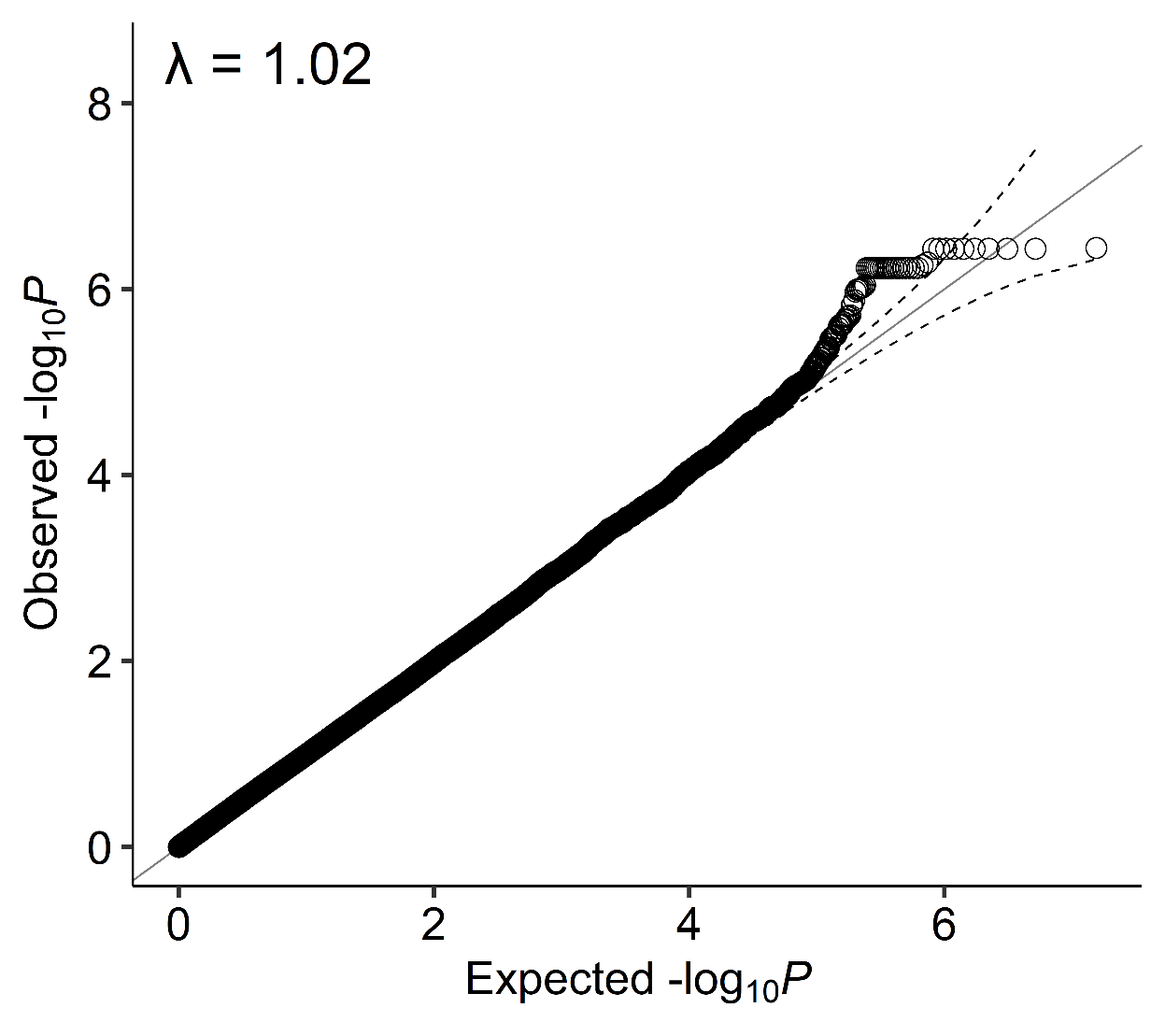


**Figure S2.** **Quantile-quantile (QQ) plot for high-titer inhibitor status in the European discovery cohort.** Association *P* values were calculated using logistic mixed-model score tests as implemented in generalized linear mixed-model association tests (GMMAT), adjusting for age, sequencing center, *F8* mutation type, hemophilia A severity, and the first 3 principal components.


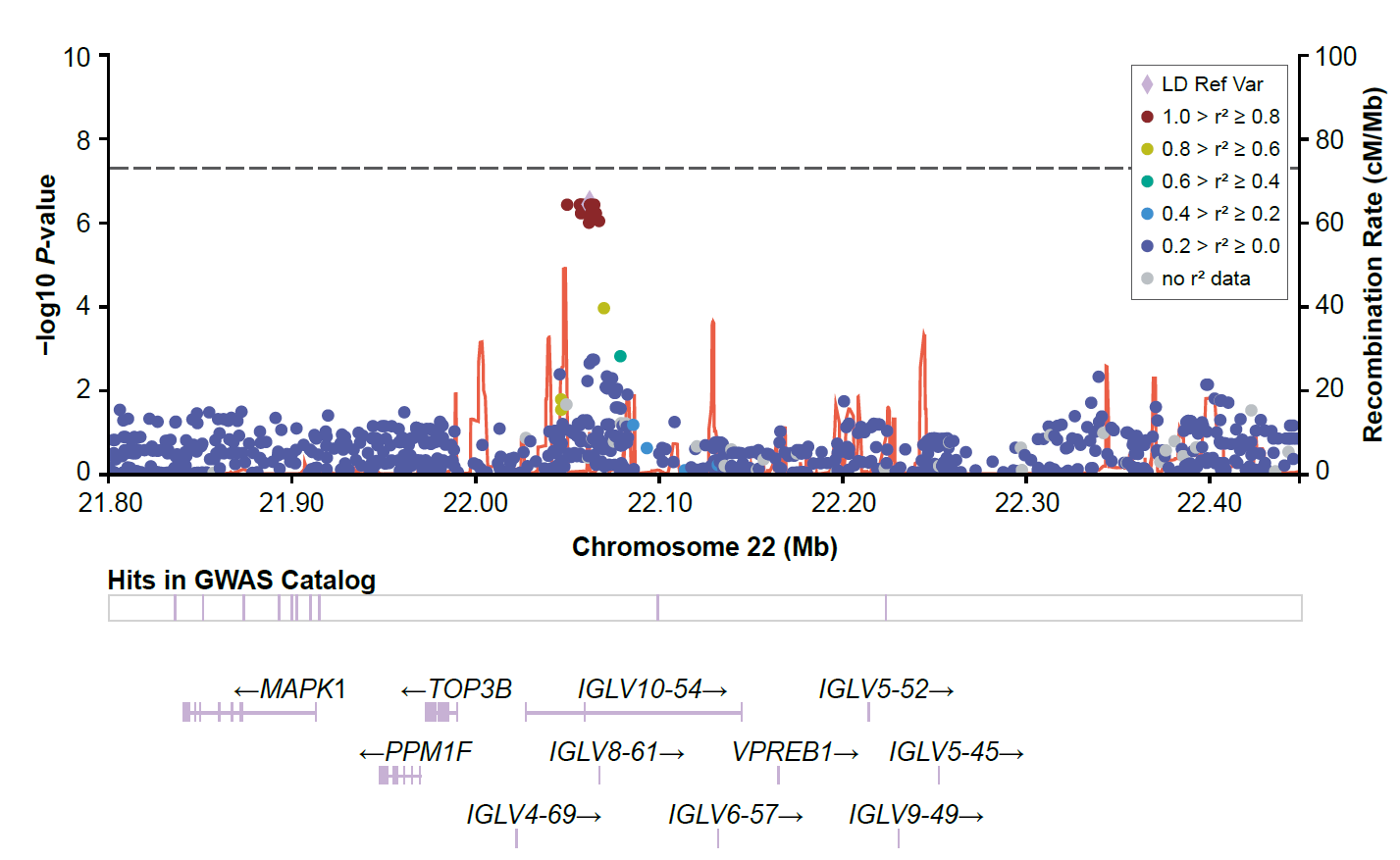


**Figure S3**. **LocusZoom plot reporting single-variant generalized linear mixed-model association tests (GMMAT) score test *P* values of high-titer inhibitors in patients with hemophilia A within the chromosome 22 *IGLV* gene cluster.** Logistic mixed models were adjusted for age, sequencing center, *F8* mutation type, hemophilia A severity, and the first 3 principal components.

GWAS, genome-wide association study; *IGLV*, immunoglobulin lambda variable; LD Ref Var, linkage disequilibrium reference variant.


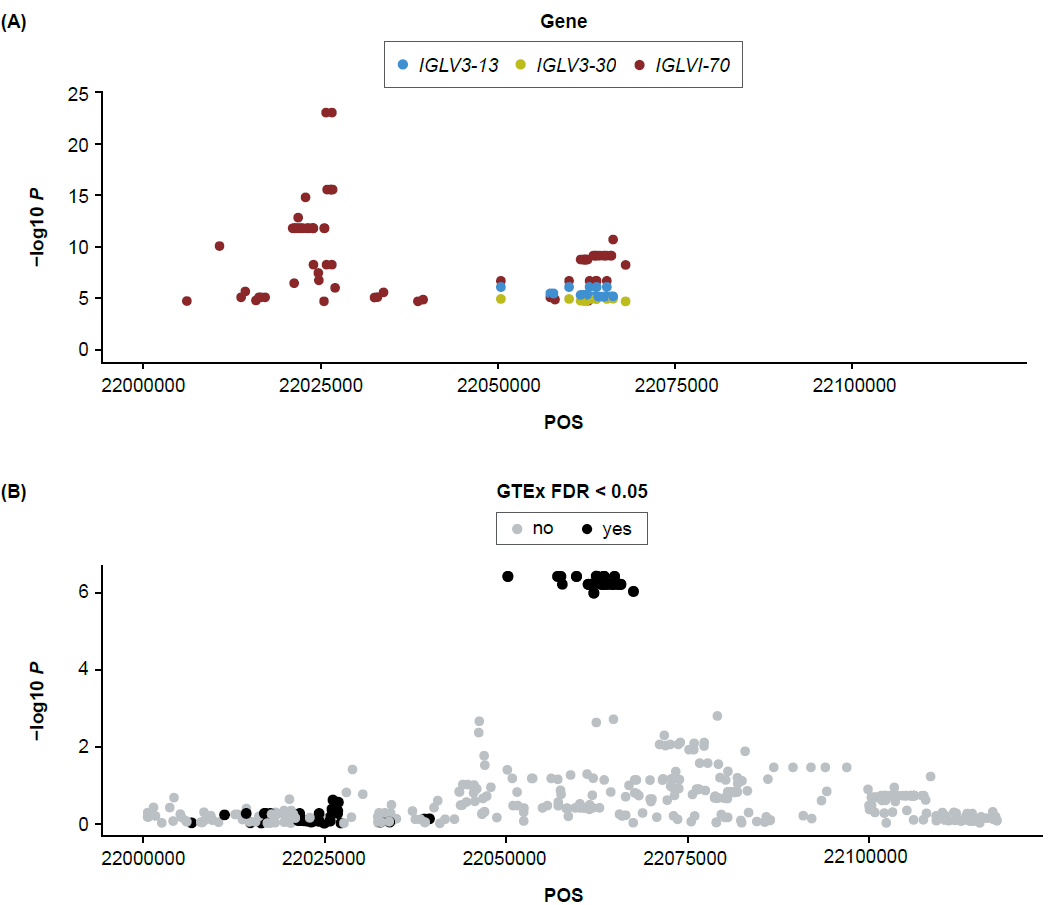


**Figure S4. Significant expression quantitative trait loci (eQTL) at the *IGLV* gene locus in minor salivary gland.** The top figure shows significant eQTLs in Genotype-Tissue Expression (GTEx) V8 minor salivary gland (False discovery rate [FDR] q-value <0.05). The bottom figure shows variants associated with high-titer inhibitors in My Life, Our Future participants of European ancestry. Highlighted dots on the bottom panel show variants overlapping with significant eQTLs in GTEx.


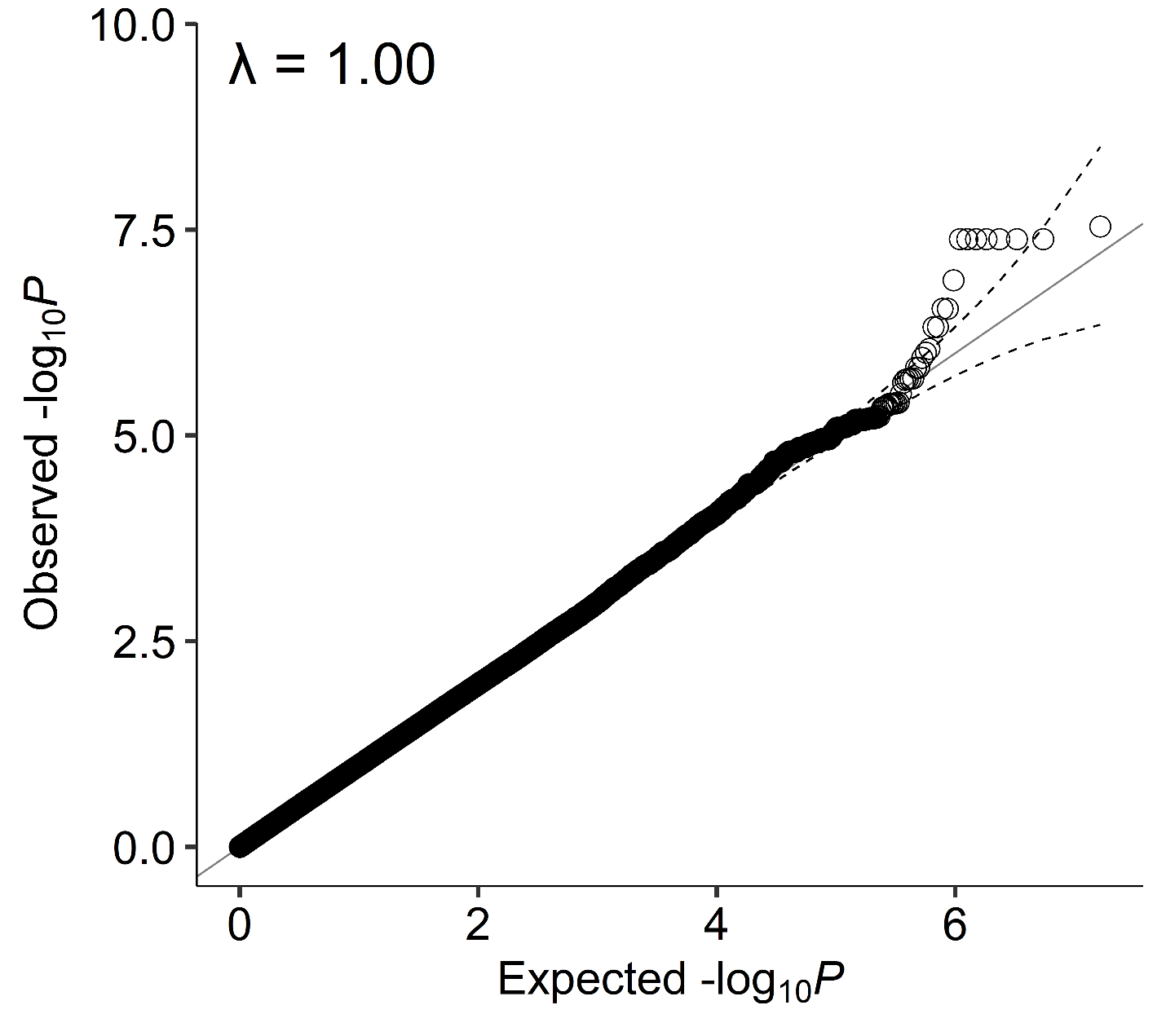


**Figure S5.** **Quantile-quantile (QQ) plot for inhibitor status in patients with intronic inversions in the European discovery cohort.** Association *P* values were calculated using logistic mixed-model score tests as implemented in generalized linear mixed-model association tests (GMMAT), adjusting for age, sequencing center, and the first 3 principal components.
