# Supplementary figures and images for: Genome-Wide Association Study and Gene-Based Analysis of Participants With Hemophilia A and Inhibitors in the My Life, Our Future Research Repository
Source: Front Med (Lausanne). 2022 Jun 23;9:903838. doi: 10.3389/fmed.2022.903838 (PMC9260508; doi:10.3389/fmed.2022.903838)

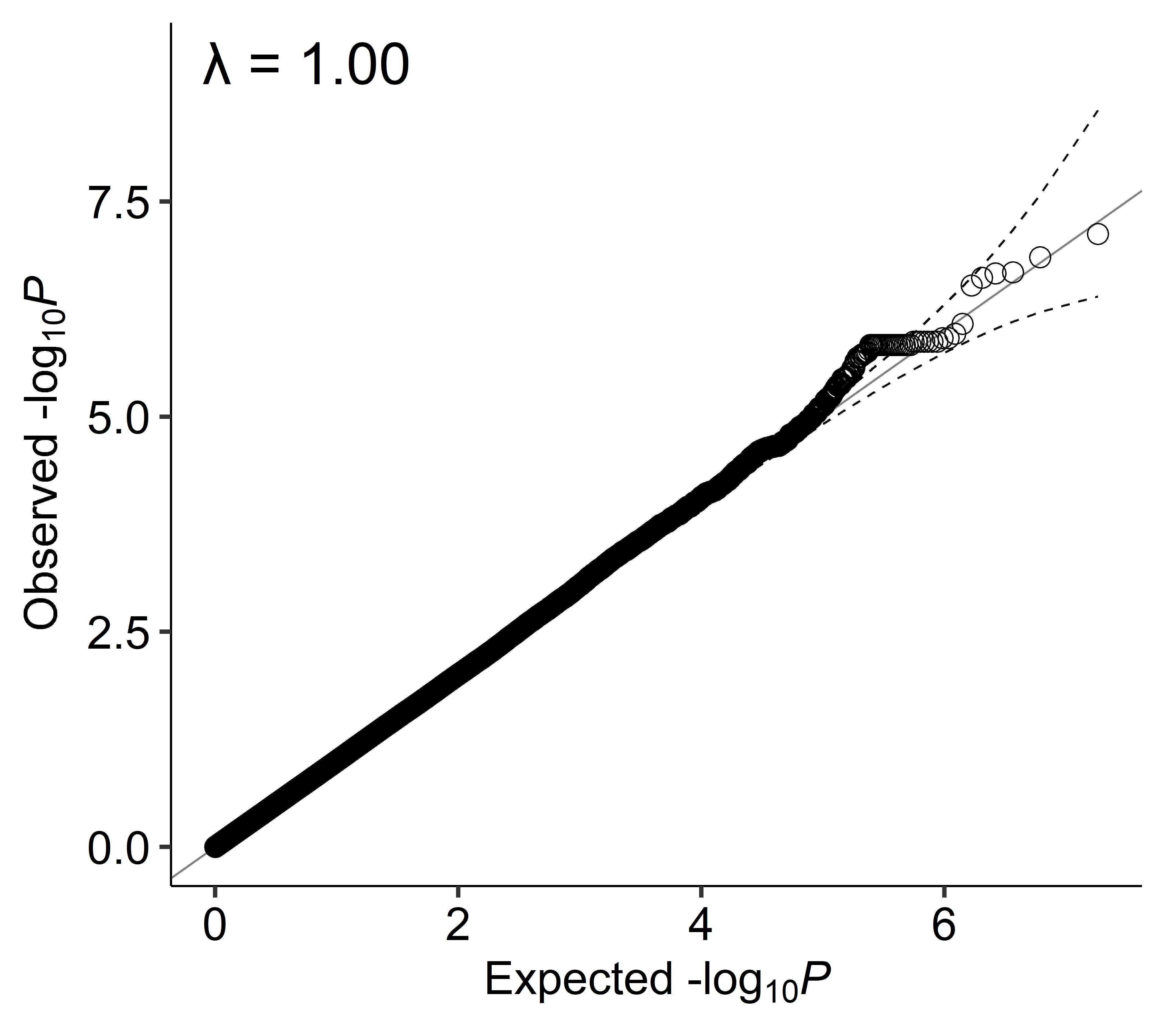

Supplement: Supplementary file 3 [file Image_1.JPEG]

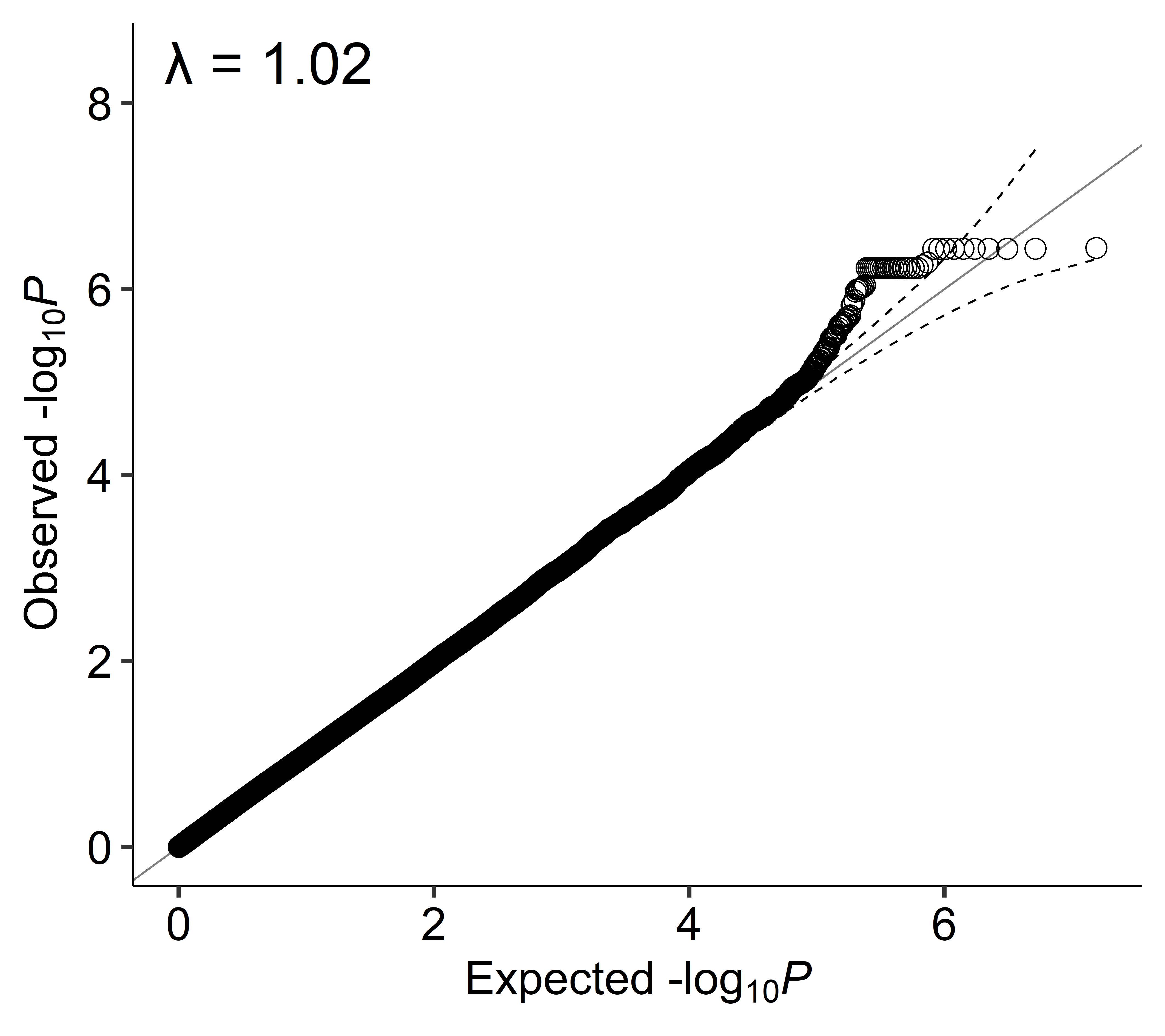

Supplement: Supplementary file 4 [file Image_2.JPEG]

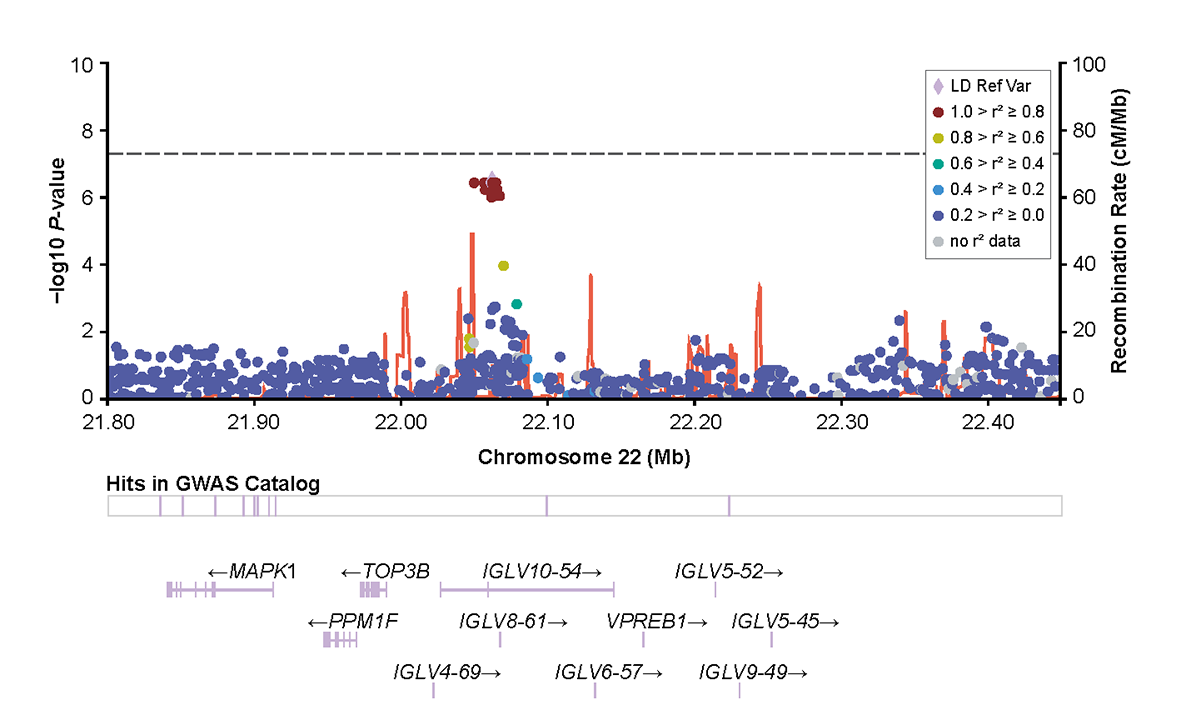

Supplement: Supplementary file 5 [file Image_3.TIF]

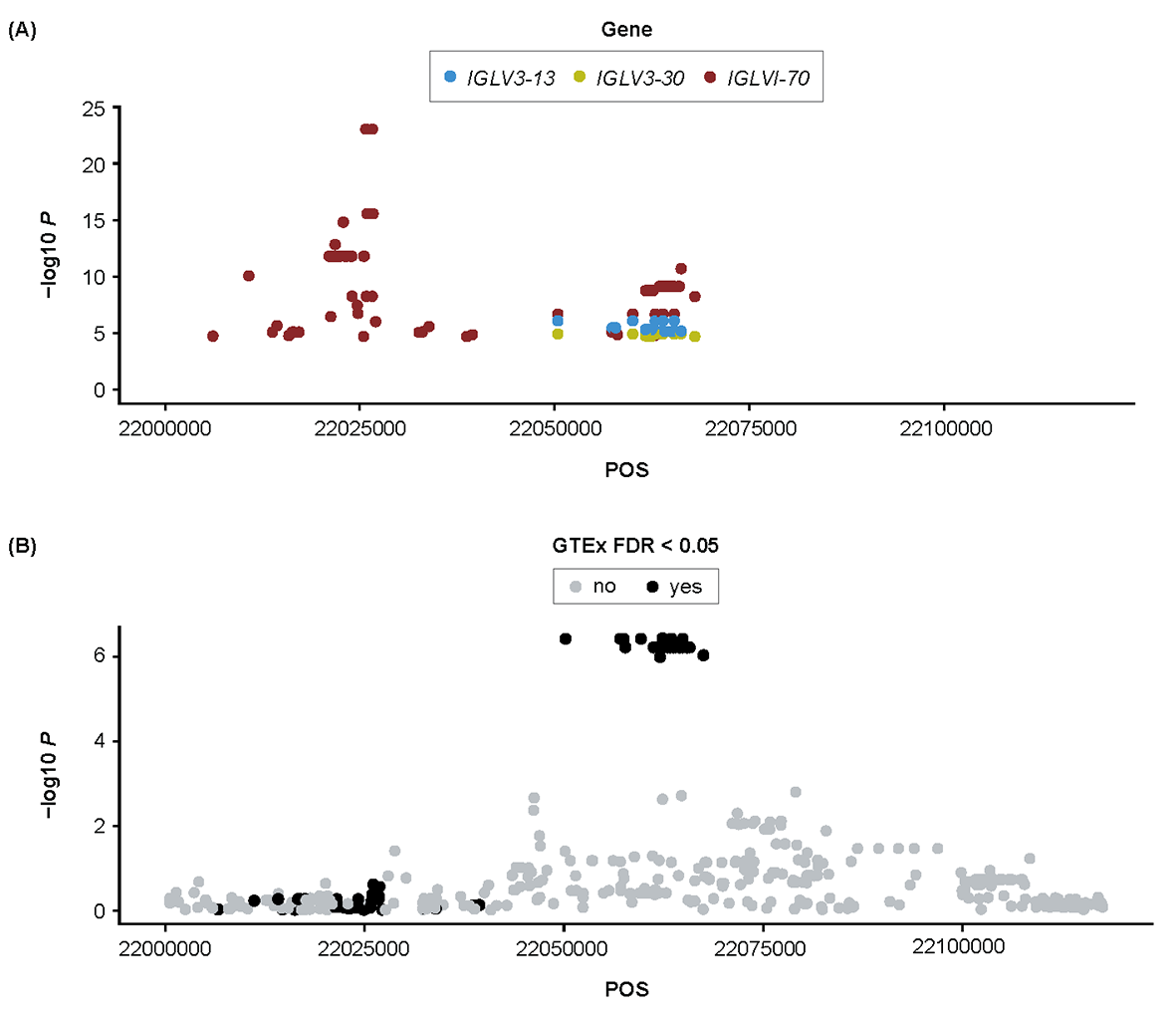

Supplement: Supplementary file 6 [file Image_4.TIF]

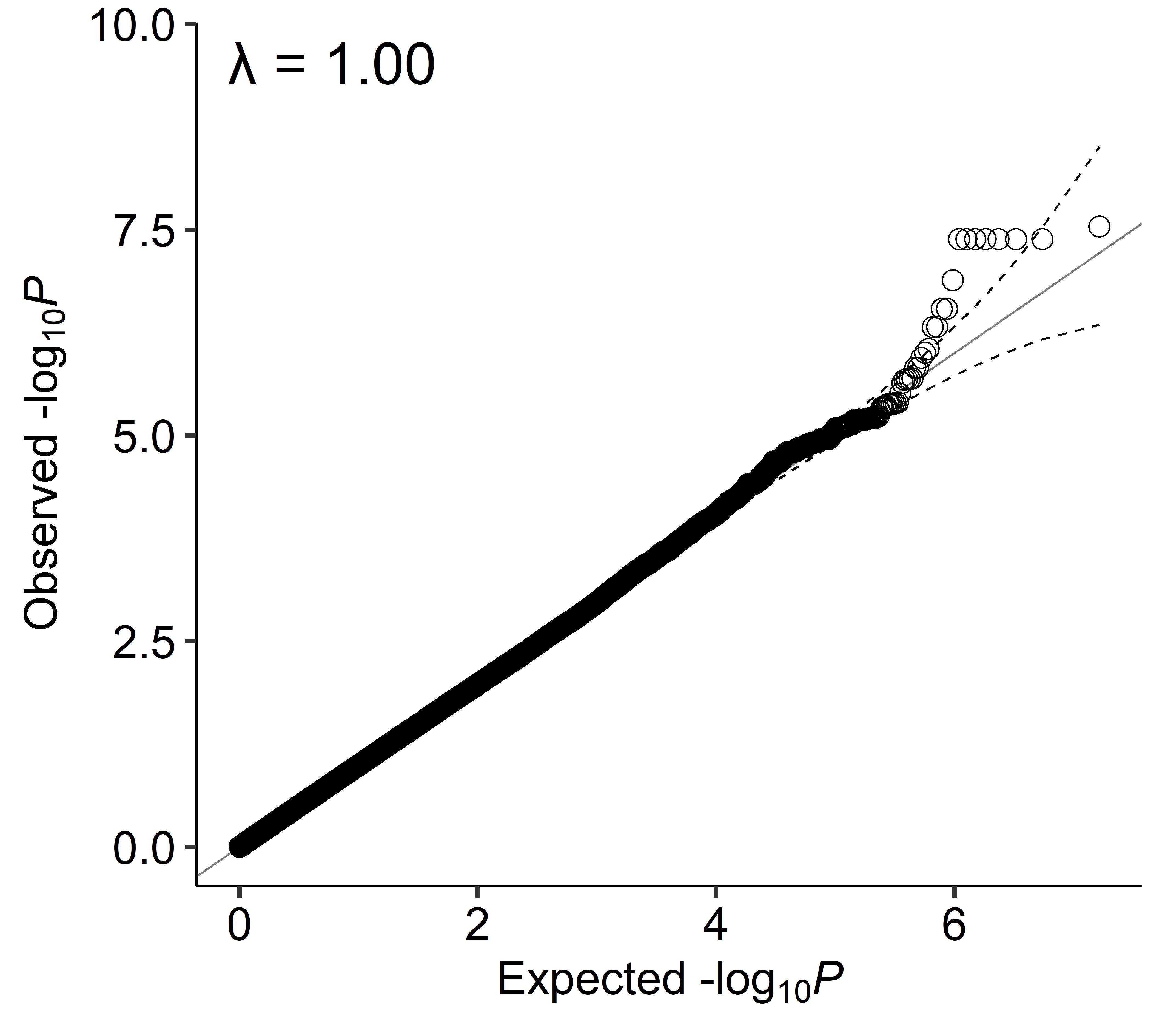

Supplement: Supplementary file 7 [file Image_5.JPEG]
